# Predicted peptide patterns from the SARS-CoV-2 proteome for MS-MS based diagnosis

**DOI:** 10.6026/97320630016477

**Published:** 2020-06-30

**Authors:** Saketh Kapoor, Pratigya Subba

**Affiliations:** 1 Stem Cells and Regenerative Medicine Center, Yenepoya Research Centre, Yenepoya (Deemed to be University), Deralakatte, Mangalore, Karnataka, Pincode-575018, India; 2 Center for Systems Biology and Molecular Medicine, Yenepoya Research Center, Yenepoya (Deemed to be University), Deralakatte, Mangalore, Karnataka, Pincode-575018, India

**Keywords:** SARS-CoV-2, COVID-19, targeted proteomics, Skyline, tryptic peptides

## Abstract

COVID-19 caused by 2019 novel coronavirus (2019-nCoV2) also known as SARS-CoV-2 has manifested globally since January 2020. COVID-19 was declared as a pandemic by the WHO and has become a serious global health concern. Real-time PCR based and antibody-based assays are being used for the clinical detection of the virus in body fluids and nasopharyngeal swabs. Antibody variability linked to viral mutations is a big concern. Hence, it is of interest to use data patterns from mass spectrometry-based platforms for the identification of SARS-CoV-2. This dataset can be used to perform targeted mass-spectrometric analysis of SARS-CoV-2 peptides. This work can be extrapolated for the detection of SARS-CoV-2 viral peptides in complex biological fluids for early diagnosis of COVID-19.

## Background

Coronavirus Disease-2019 (COVID-19), the most recent global pandemic that originated in Wuhan, China, is caused by the viral pathogen Severe Acute Respiratory Syndrome Coronavirus-2 (SARS-CoV-2). Clinical symptoms associated with the viral infection include fever, respiratory distress and pneumonia that may also lead to death [1]. SARS-CoV2 is an enveloped virus with a 30Kb single-stranded positive-sense RNA genome [2]. The genomic organization (5' to 3') includes six major ORFs: ORF 1a and ORF1b that together encode for sixteen non-structural proteins (Nsp1-16), followed downstream by structural proteins S (Spike), E (Envelope), M (Membrane), N (Nucleocapsid) [1][3]. Additionally, there are 9 accessory proteins (ORF 3a, ORF 3b, ORF6, ORF7a, ORF7b, ORF8, ORF9a, ORF9b, ORF10). SARS-CoV-2 genome shared highest identity (∼96%) with a horseshoe bat (Rhinolophus affinis) virus RaTG13 [1]. Among the six previously reported coronaviruses that infect humans (SARS-CoV, MERS-CoV, NL63, HKU1, OC43 and 229E), SARS-CoV-2 has highest nucleotide sequence identity (∼80%) with SARS-CoV [3]. The S protein shows highest divergence compared to RaTG13 within the receptor-binding domain (RBD) and insertion of polybasic furin cleavage site (681PRRA684) between S1 and S2 subunits [2]. The RBD region of SARS-CoV-2 S protein has higher (97%) amino acid identity with a pangolin virus leading to speculations about pangolins as intermediate SARS-CoV2 reservoirs [2]. SARS-CoV-2 (like SARS-CoV) utilizes the host ACE2 receptor to gain cellular entry [1]. Tissue specific expression patterns of ACE2 receptor showed enrichment in human alveolar epithelial cells, human gut epithelium and various other tissues [4]. Currently qRT–PCR is the mostly reliable and widely used technique to detect SARS-CoV2 transcripts [1]. Together, with understanding differentially regulated host proteins in response to SARS-CoV-2 infection, tandem mass spectrometry has been used to detect the viral peptides in infected Vero E6 cells [5- [6]. Although shotgun proteomics allows large-scale identification and quantification of proteins, the wide dynamic range of the human proteome does not allow detection of low abundant pathogen-derived peptides in biological samples. Targeted proteomics is a hypothesis-driven approach that allows selective monitoring of proteotypic peptides, which represent the protein(s) of interest [7]. In this study, we generated the theoretical proteotypic peptides of SARS-CoV-2 and their corresponding transitions using the open-source Skyline [8]. The data can be used to develop targeted proteomics assays for the specific detection and/or quantification of SARS-CoV2 peptides in complex clinical sample types.

## Materials and Methods:

SARS-CoV-2 proteome was downloaded from RefSeq in FASTA file format. The file was imported to Skyline and theoretical tryptic peptides were generated [8]. Peptide settings were adjusted to: digestion trypsin [KR] [P], maximum missed cleavage 1, peptide length 8-25, modification carbamidomethyl (C) and included heavy isotopes. Transition settings were adjusted to: precursor charge 2, ion charges 1, ion type b and y, product ion selection was set from m/z>precursor to 3 ions. The output data were exported from Skyline in .csv format and analyzed in Microsoft Excel.

## Results and discussion:

Targeted proteomic workflow prioritizes the identification of selected peptides(s) of interest in complex biological samples [7]. The selected (proteotypic) peptides must be detectable in a mass spectrometer and must be specific to the organism. We downloaded a non-redundant set of SARS-CoV-2 protein sequences from RefSeq and generated a list of their theoretical tryptic peptides (Table 1). We considered both, 'b' and 'y' ions. As the downstream experiments may also require the 'spiking in' with heavy isotope (Lys and Arg) versions of the selected peptides into complex tryptic digests of biological samples, we included the 'light' and 'heavy' peptides in our analysis (Table 1). The length of the peptides varied from 8 to 25 amino acids. A total of 1744 theoretical light and heavy tryptic peptides were generated from the entire SARS-CoV-2 proteome, which led to the generation of 21,023 transitions. A partial list of proteotypic peptides along with their corresponding transitions are listed in Table 1. The number of tryptic peptides varied from 655 in Orf1ab polyprotein (YP_009724389.1) to 3 peptides in Nsp11 (YP_009725312.1). Among the non-redundant set of tryptic peptides, 101 contained a single trypsin missed cleavage site and 562 contained carbamidomethyl (C) residues. Although the selection of such peptides requires a careful scrutiny during mass spectrometric data acquisition, we considered these peptides for our analysis as we aimed at generating maximum number of candidate peptides for downstream targeted proteomics analysis.

As expected, we observed highest number of peptides of Orf1ab polyprotein (YP_009724389.1) and Orf1a polyprotein (YP_009725295.1). Orf1ab polyprotein contained 655 non-redundant peptides that yielded 7365 transitions. Orf1a polyprotein contained 362 non-redundant peptides that yielded 4079 transitions. Comparative analysis of the non-redundant lists of peptides of these 2 proteins revealed a single terminal peptide (EPMLQSADAQSFLNGFAV) that was unique to Orf1a polyprotein. These polyproteins encode components of the viral replicase and have 94.4% amino acid sequence identity with the corresponding genes in SARS-CoV [1].The synthesized viral polyproteins are subject to various host and viral proteases at specific sites [3]. As these proteases regulate an important step in viral replication, they serve as lucrative targets for designing anti-viral drugs. A recent in silico study screened 10 FDA approved drugs against SARS-CoV-2 protease [9]. RNA-dependent RNA polymerase (Nsp12) bears 96.2% sequence identity with that of RaTG13 [1]. This protein is the target for a widely used FDA approved drug remdesivir also projected as a drug to treat COVID-19 [10]. Non-structural protein Nsp11 is only 13 amino acids long due to which it did not fit our Skyline peptide filter criteria. We therefore manually generated the proteotypic peptides for this protein. We observed a single proteotypic peptide along with 6 transitions (Table 1).

Viral structural proteins are major targets for designing or repurposing antiviral drugs. The S protein (YP_009724390.1) performs 2 important functions: enables virus binding to host ACE2 receptor using RBD and fusion of viral and host membranes. Although SARS-CoV-2 genome shares highest sequence identity with the bat virus RaTG13, the S protein showed highest percentage of dissimilarity [2]. This protein may also enable us to distinguish SARS-CoV-2 from SARS-CoV. This protein was reported to interact with the host protein palmitoyltransferase ZDHHC5 that led to speculations about palmitoylation of S protein [11]. For this protein, we observed 102 tryptic peptides and 1170 transitions (Table 1). The E protein is a membrane protein involved in viral assembly and propagation [12]. We observed 5 non-redundant peptides for this protein that yielded 22 transitions. SARS-CoV-2 E protein has been reported to interact with host bromodomain-containing proteins BRD2 and BRD4 [11]. Interestingly, the BRD binding region of E protein bears structural similarity with acetylated histones leading to speculations about the BRD-E protein interactions is altering host gene expression. Structural protein M (YP_009724393.1) has been previously reported to interact with E and S proteins in the host cell budding compartment protein and is involved in viral assembly [12]. Along with homotypic interactions, M protein also interacts with multiple other viral proteins such as viral N protein and accessory proteins 3a and 7a. For M protein we observed 16 non-redundant peptides corresponding to 188 transitions. Interactions between M and N proteins are required for packaging of the viral genetic material [12]. SARS-CoV structural proteins E, M and N have been reported to be secreted into the media and are required for the production and release of virus-like particles [13]. For N protein (YP_009724397.2), we observed 54 non-redundant tryptic peptides along with 600 transitions. The secretory nature of these proteins (E, M and N) makes them lucrative targets for detecting the presence of virus using biological fluids (Supplementary Table 1 in Excel file format).

## Conclusions:

In this study, we describe a workflow using predicted data patterns from SARS-CoV2- tryptic peptides for potential application in diagnosis. This data will serve as a resource for the selection of candidate SARS-CoV-2 peptides for conducting targeted mass spectrometry-based experiments in biological samples. The experiments can be optimized using synthetic peptides to choose the "best flyer" in the mass spectrometer. Additionally, the synthetic stable-isotope labelled peptides can be used for absolute quantitation of selected viral peptides.

## Figures and Tables

**Table 1:** Partial list of proteotypic peptides along with their corresponding transitions.

Protein ID	Peptide sequence	Precursor ion	Product ion	Ion Name
YP_009724389.1	AIDGGVTR.light	394.71	414.19	b5
YP_009724389.1	AIDGGVTR.heavy	399.72	414.14	b5
YP_009724390.1	ASANLAATK.light	423.73	457.24	b5
YP_009724390.1	ASANLAATK.heavy	427.74	625.37	y6
YP_009724391.1	DATPSDFVR.light	504.24	536.28	y4
YP_009724391.1	DATPSDFVR.light	504.24	587.23	b6
YP_009724395.1	HVYQLRAR.light	521.79	528.25	b4
YP_009724395.1	HVYQLRAR.light	521.79	641.34	b5
YP_009724397.2	GGSQASSR.light	375.18	401.17	b5
YP_009724397.2	GGSQASSR.light	375.18	420.22	y4
YP_009724397.2	GGSQASSR.light	375.18	401.17	b5
YP_009724397.2	GGSQASSR.heavy	380.18	645.31	y6
YP_009725309.1	LISMMGFK.light	463.74	482.24	y4
YP_009725309.1	LISMMGFK.heavy	467.75	576.28	b5

**Figure 1:**
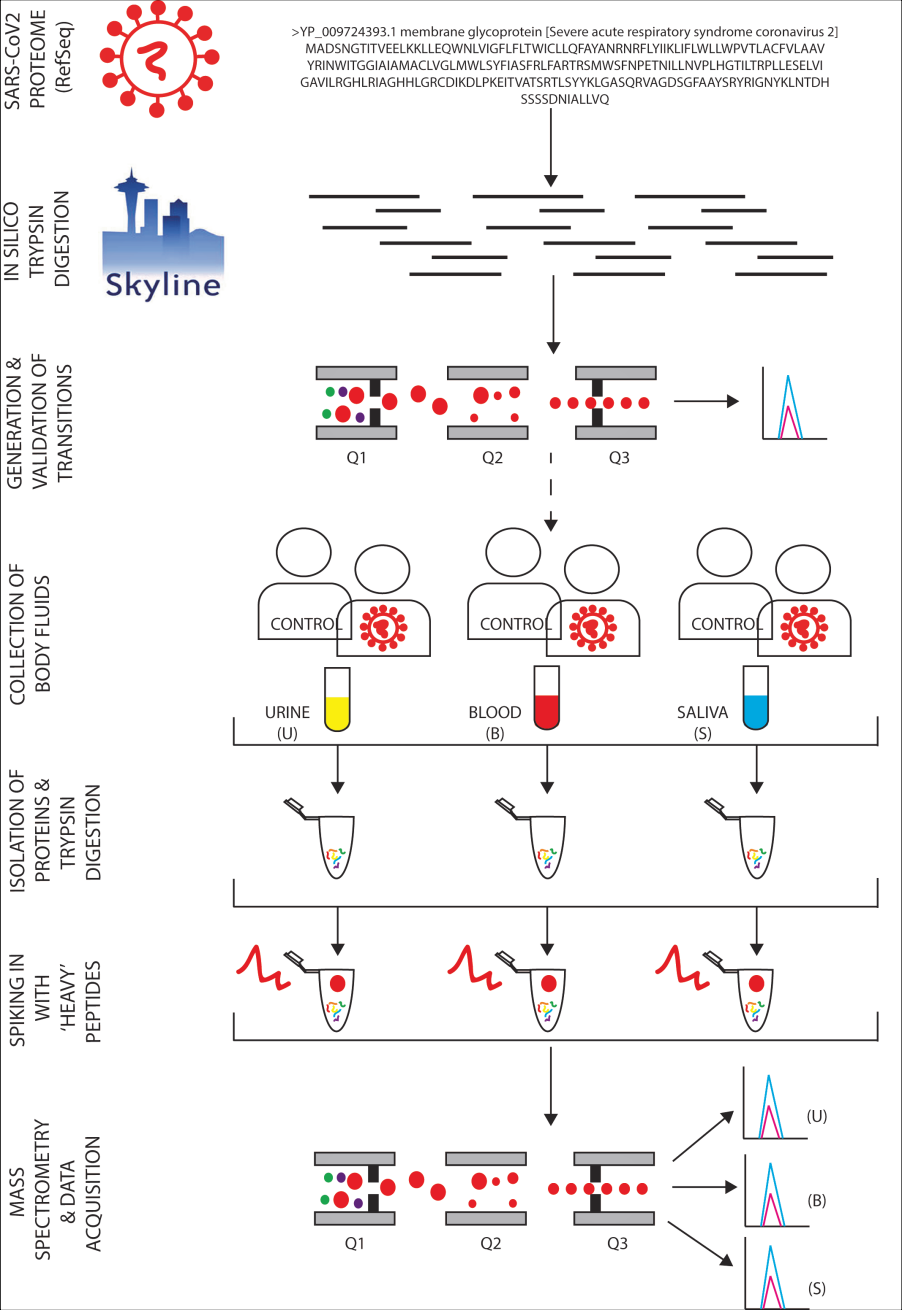
Workflow depicting the predicted data patterns from SARS-CoV-2 tryptic peptides generated using Skyline for potential application in diagnosis.

## References

[1] Zhou P (2020). Nature..

[2] Zhang YZ (2020). Cell..

[3] Wu F (2020). Nature..

[4] Zhang H (2020). Intensive Care Medicine..

[5] Bojkova D (2020). Nature..

[6] Davidson AD (2020). bioRxiv..

[7] Borras E (2017). Proteomics..

[8] MacLean B (2010). Bioinformtaics..

[9] Odhar AH (2020). Bioinformation..

[10] Wang M (2020). Cell Research..

[11] Gordon DE (2020). bioRxiv..

[12] Schoeman B (2019). Virology Journal..

[13] Siu YL (2008). Journal of Virology..

